# Chronic Alcohol Ingestion Delays T Cell Activation and Effector Function in Sepsis

**DOI:** 10.1371/journal.pone.0165886

**Published:** 2016-11-18

**Authors:** Lindsay M. Margoles, Rohit Mittal, Nathan J. Klingensmith, John D. Lyons, Zhe Liang, Mara A. Serbanescu, Maylene E. Wagener, Craig M. Coopersmith, Mandy L. Ford

**Affiliations:** 1 Division of Infectious Diseases, Emory University, Atlanta, GA, United States of America; 2 Department of Surgery, Emory University, Atlanta, GA, United States of America; 3 Emory Transplant Center, Emory University, Atlanta, GA, United States of America; 4 Emory Critical Care Center, Emory University, Atlanta, GA, United States of America; University of Florida, UNITED STATES

## Abstract

Sepsis is the leading cause of death in intensive care units in the US, and it is known that chronic alcohol use is associated with higher incidence of sepsis, longer ICU stays, and higher mortality from sepsis. Both sepsis and chronic alcohol use are associated with immune deficits such as decreased lymphocyte numbers, impaired innate immunity, delayed-type hypersensitivity reactions, and susceptibility to infections; however, understanding of specific pathways of interaction or synergy between these two states of immune dysregulation is lacking. This study therefore sought to elucidate mechanisms underlying the immune dysregulation observed during sepsis in the setting of chronic alcohol exposure. Using a murine model of chronic ethanol ingestion followed by sepsis induction via cecal ligation and puncture, we determined that while CD4+ and CD8+ T cells isolated from alcohol fed mice eventually expressed the same cellular activation markers (CD44, CD69, and CD43) and effector molecules (IFN-γ, TNF) as their water fed counterparts, there was an overall delay in the acquisition of these phenotypes. This early lag in T cell activation was associated with significantly reduced IL-2 production at a later timepoint in both the CD4+ and CD8+ T cell compartments in alcohol sepsis, as well as with a reduced accumulation of CD8^dim^ activated effectors. Taken together, these data suggest that delayed T cell activation may result in qualitative differences in the immune response to sepsis in the setting of chronic alcohol ingestion.

## Introduction

Alcohol use disorders are prevalent in the United States, with recent epidemiologic data based on the American Psychiatric Association’s clinical diagnostic criteria (DSM-5) estimating twelve month and lifetime prevalence of the condition to be 13.9% and 29.1%, respectively [1, 2]. Acute and chronic alcohol use have long been known to be associated with increased susceptibility to infections such as bacterial pneumonia, tuberculosis, viral hepatitis, and HIV, as well as increased morbidity from these infections [3]. Beyond these associations with specific infections, clinical studies have shown that chronic alcohol users are more susceptible to developing sepsis and septic shock and have prolonged hospital stays and increased mortality [4] from this syndrome.

While the poor clinical outcomes observed in patients with a chronic alcohol use history has been known for sometime, the specific mechanisms responsible for this condition of relative immune compromise are not fully understood. Some of the suppressive effects of chronic alcohol use on the innate immune system have been well-delineated including decreased tissue recruitment and phagocytic function of neutrophils, decreased number and co-receptor function of dendritic cells (DCs), and decreased cytotoxic effect of natural killer (NK) cells [3]. In contrast to its suppressive effects on some immune cells, chronic alcohol ingestion is also associated with elevated baseline serum levels of inflammatory cytokines such as TNF, IL-1, IL-6, and IL-12 [5] [3]. With regard to the adaptive immune system, several studies have examined the Th1/Th2 profiles of chronic alcohol users, which predominantly point toward Th2 skewing with increased antibody and autoantibody production [5]. Previous murine studies throughout the 1990s demonstrated the Th2 skewing resulting from chronic ethanol administration was associated with decreased T cell production of IFN-γ, increased IL-4, and down-regulation of IL-12, with preservation of T cell proliferation and IL-2 production. These deficits were subsequently localized to the APC and shown to be rescued by administration of IL-12 [6]. Similarly, in pathogen-specific infection models including *Klebsiella pneumoniae* pneumonia and Hepatitis C infection, alcohol ingestion decreased Th1 responses and was hypothesized to enhance Th2 immune response associated with impaired IL-2 secretion from DCs and T cell suppression of IL-12 by macrophages and DCs [3] [7] [6, 8]. Several studies have shown impaired CD8+ T cell function in the setting of chronic ethanol ingestion through demonstration of delayed viral clearance [3]. In contrast, increased frequency of activated CD4+ T cells in the setting of chronic ethanol exposure has also been reported in the literature [9] [10], though the mechanistic consequences of this finding are not clearly defined. Some recent studies provide more nuanced description of changes in T cell subsets due to chronic ethanol. Partlet et al. demonstrated selective loss of epidermal and dermal regulatory T cells, specifically due to reduced proliferation and increased apoptosis, as well as decreased expression of JAML and CD69 on dendritic epidermal T cells following ex vivo stimulation and impaired IL-17 responses[11]. Additionally, in a murine model of Influenza A virus (IAV) infection following ethanol ingestion, ethanol was associated with increased mortality, increased viral titers, and decreased IAV-specific CD8 T cells with reduced proliferation, IFN-γ production and degranulation induced target cell lysis[12].

As our understanding of T cell dysregulation associated with sepsis has expanded in recent years, it is unknown if these ethanol-induced immune changes are conserved in sepsis or if there are unique immunological characteristics that emerge in this setting. The key features of immune dysregulation due to sepsis have become better characterized over the past decade, with the increasingly accepted paradigm of a brief hyperinflammatory response followed by a nearly concomitant and prolonged immune suppressive stage which results in lack of eradiation of the septic focus and increased susceptibility to subsequent infections by less virulent pathogens [13]. Among the multitude of immune deficits in sepsis, marked T cell apoptosis has been a well-characterized phenomenon for many years, and more nuanced descriptions of many aspects of T cell biology have been published recently [14–16]. Numerous animal and human studies have begun to unravel the immunopathology of sepsis associated with widespread lymphocyte apoptosis, down regulation of major histocompatibility complex expression and decreased antigen presentation, and reduced T cell receptor diversity [15–18]. In addition to cell loss, T cells have been shown to exhibit increased co-inhibitory receptor expression such as programmed cell death receptor-1 (PD-1), cytotoxic T lymphocyte antigen-4 (CTLA-4), and B and T lymphocyte attenuator (BTLA), and a shift toward production of immune suppressive cytokines (eg, IL-10) [17–19]. The precise nature of these complicated pathways are still being elucidated as we enter into an era of investigation into immunomodulatory therapies for sepsis, many of which aim to reverse this prolonged immunosuppressive stage of the syndrome. Thus far, even less is known about these pathways in either overtly immunosuppressed patients or functionally immunosuppressed states such as advanced age or chronic ethanol exposure. With T cell modulating agents such as anti-PD-1 antibody and IL-7 currently under investigation for therapeutic use in sepsis, it is important that detailed immunophenotyping be performed to determine whether these agents may perform differently in the setting of comorbidities which confer a unique baseline immune changes, such as chronic ethanol exposure.

Our lab has previously published data demonstrating that the setting of chronic alcohol ingestion results in a significant increase in mortality following sepsis [20]. In a similar rat model of polymicrobial sepsis using intraperitoneal injection of fecal material, Barros et al. recently demonstrated a dose-dependent increase in mortality due to sepsis after chronic ethanol ingestion [21]. We hypothesize that this increased mortality is the result of the confluence of multiple immune deficits present in the conditions of both chronic alcohol ingestion and sepsis, but the identities of the key mediators of this effect are not yet known. Here, in an effort to enhance understanding of the observed increased mortality from sepsis in this setting, we sought to determine the impact of chronic alcohol ingestion on T cell expansion, activation, and effector function during sepsis.

## Materials and Methods

### Animals and alcohol feeding protocol

**Fig 1:**
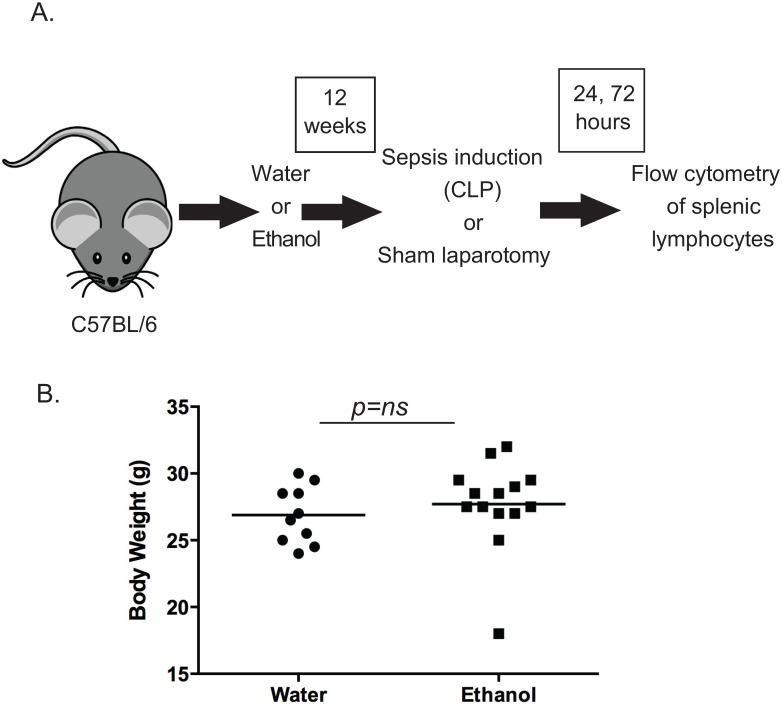
Experimental design. A, 4–6 week old C57BL/6 mice were provided either ethanol or water for 12 weeks followed by sepsis induction via CLP or sham laparotomy. Spleens were harvested at 24 and 72 hours for flow cytometric analysis of lymphocytes. B, Body weights were not different between water-fed and alcohol-fed mice at the end of the 12-week period.

For the chronic alcohol ingestion protocol, six-week old male C57BL/6 mice were purchased from Jackson Laboratories (Bar Harbor, ME) and housed in an animal facility at Emory University. Mice were allowed to acclimate to the facility for one week before being randomized to receive either water or alcohol. Alcohol-fed mice were given increasing concentrations of ethanol from 0%-20% (volume/volume) over a two-week period followed by 10 weeks of 20% ethanol (Fig 1A). Control animals drank water for the same time period [20, 22, 23]. All animals had free access to laboratory chow throughout. At the end of the alcohol feeding protocol, no significant differences in body weight were observed between the water and alcohol fed groups (Fig 1B). All experiments were performed in accordance with the National Institutes of Health Guidelines for the Use of Laboratory Animals and were approved by the Institutional Animal Care and Use Committee at Emory University School of Medicine (protocol DAR 2002473-090316BN).

### Sepsis model

Sepsis induction was performed through cecal ligation and puncture (CLP) as previously published [24]. Mice were anesthetized with isofluorane, followed by a small midline incision and externalization of the cecum. The cecum was ligated distal to the ileocecal valve with care to avoid intestinal obstruction. The cecum was then punctured twice with a 30g needle and a small amount of stool extruded prior to returning the contents to the abdominal cavity. The incision was then closed in layers. Sham control animals underwent an identical procedure but the cecum was not punctured. All mice received buprenorphine preoperatively for pain relief as well as postoperative 1ml of normal saline for replacement of insensible losses and antibiotics (ceftriaxone 25mg/kg and metronidazole 12.5mg/kg, subcutaneously) up to four doses every 12 hours after surgery until euthanasia. The experimental design is illustrated in Fig 1.

### Flow cytometric analysis

Splenocytes were harvested at 24 hours and 72 hours after sepsis induction and processed into single cell suspensions for surface staining of cellular activation markers with CD4-Pacific Blue, CD8a-APC-Cy7, CD44-FITC, CD69-PECy7, CD43-APC. AccuCheck counting beads (Life Technologies) were used to determine cell numbers. Data were acquired on a BD Bioscience LSRII flow cytometer.

### Ex vivo restimulation and intracellular cytokine staining

Production of intracellular cytokines IFN-γ, IL-2, and TNF after ex vivo stimulation was measured at 24 hours and 72 hours after sepsis induction with an intracellular cytokine staining kit (BD Biosciences) per the manufacturer’s instructions. Splenocytes were harvested and stimulated ex vivo with phorbol 12-myristate 13-acetate (PMA, 30 ng/mL) and ionomycin (400 ng/mL) in the presence of Brefeldin A for five hours. After incubation, cells underwent surface staining with CD4-Pacific Blue and CD8a-Pacific Orange (BD Bioscience, San Jose, CA) followed by intracellular staining with IFN-γ-Alexa 700 (eBioscience, San Diego, CA), TNF-PECy7, and IL-2-AF488 (BD Bioscience, San Jose, CA). Data were acquired on a BD Bioscience LSRII flow cytometer. For analysis, staining, doublets were excluded and a lymphocyte gate drawn based on size and complexity. Lymphocytes were then divided into CD4+ and CD8+ populations. Each lymphocyte group was then analyzed for frequency of cytokine producing cells based on intracellular staining for IL-2, TNF, and IFN-γ. Gates for positivity were drawn using the water-fed sham unstimulated population as a baseline value.

### Serum cytokine analysis

Whole blood was collected from water-fed- and EtOH-fed animals at 24 h following CLP or sham surgery and then centrifuged at 10,000 RPM for 10 minutes. The supernatant from each was then collected and analyzed for cytokine concentrations using a 6-plex cytokine bead array according to manufacturer instructions (Bio-Rad Laboratories, Hercules, CA).

### Statistical analysis

All flow cytometric data were analyzed by Flow Jo X software (Tree Star, San Carlos, CA). All data were analyzed using the statistical software program Prism 5.0 (GraphPad, San Diego, CA) and are presented as (mean +/- SEM). For groups larger than seven, data were tested for Gaussian distribution using the Shapiro-Wilk normality test. For multiple group comparisons with normal distributions, one-way ANOVA followed by Tukey post-test was used. If data were not normally distributed or too small to be determined, Kuskal-Wallis test with a Dunn’s test for multiple comparisons was used. A p value of <0.05 was considered statistically significant.

## Results

In order to investigate the effect of chronic alcohol ingestion on the lymphocyte compartment in the setting of sepsis we subjected mice to a 12 week ethanol feeding protocol followed by sepsis induction by CLP (Fig 1A). Body weights were not different between water-fed and alcohol-fed mice at the end of the 12-week period (Fig 1B). To differentiate between the individual effects of either alcohol or sepsis, we utilized four experimental groups: water-fed with sham laparotomy, alcohol-fed with sham laparotomy, water-fed with CLP, alcohol-fed with CLP.

**Fig 2:**
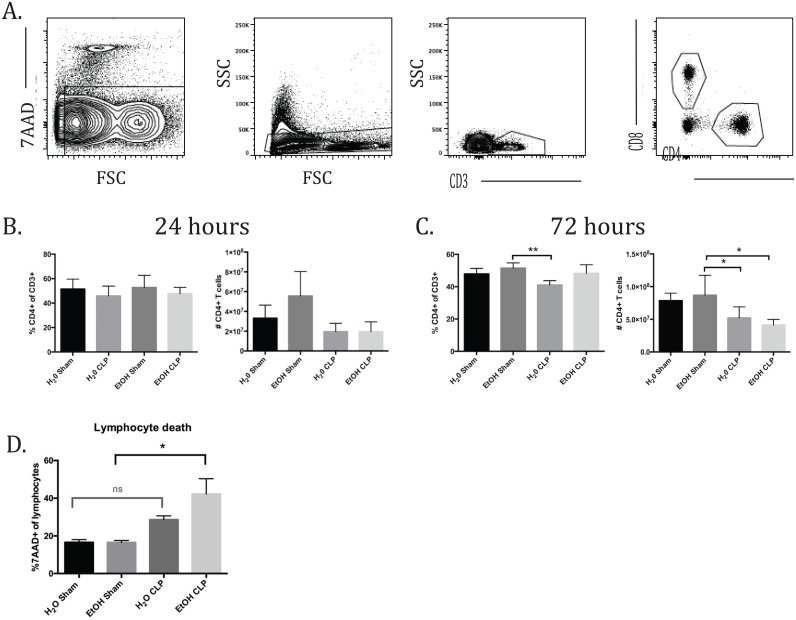
CD4+ T cell frequencies and counts. A) Representative flow plots and gating strategy. B) There were no differences in CD4 frequencies or counts at 24h. C) At 72h, there were no relevant differences in CD4 frequency. A significant decrease in cell count was seen between alcohol sham and alcohol CLP (8.7x10^7^±1.3x10^7^ vs 4.1x10^7^±3.8x10^6^, p = 0.03). n = 6-9/group.

In order to assess the combined impact of ethanol consumption and sepsis on T cell dysregulation, whole spleens were processed and prepared with surface staining markers as described above. For each analysis, the gating began with exclusion of doublets followed by identification of the live cell population based on 7AAD staining. A lymphocyte gate was then drawn based on size and complexity (forward scatter by side scatter). CD3+ cells were first identified and then further divided into CD4+ and CD8+ populations (Fig 2A). For both groups of T cells, the populations were analyzed for overall expression of CD44, CD69, and CD43. CD4+ and CD8+ cells were divided into CD44^hi^ or CD44^lo^ populations to further differentiate expression of CD69 and CD43 in these subgroups.

### Alcohol exposure delayed the kinetics of CD69 expression on naïve CD4+ T cells and prolonged CD69 expression on memory CD4+ T cells

These studies failed to show any changes in CD4+ T cell frequency 24 hours after sepsis or sham laparotomy. There was a trend toward increased absolute number of CD4+ T cells in the EtOH sham group, which appears reduced in the setting of sepsis, but this did not reach statistical significance due to intragroup variability (Fig 2B). At 72 hours, there were no significant changes in CD4+ frequency due to the additional of alcohol alone or in synergy with sepsis as seen by comparison of the H_2_O CLP and EtOH CLP groups (Fig 2B and 2C). We observed a significant decrease in the absolute number of CD4^+^ T cells in the EtOH CLP group compared with EtOH sham at 72h post-CLP, while the decrease in CD4^+^ T cells in the water-fed CLP animals relative to water-fed sham controls failed to achieve significance. Similarly, a significant increase in 7-AAD^+^ apoptotic lymphocytes was observed in EtOH-CLP animals relative to EtOH sham controls, while the increase in apoptotic lymphocytes in water-fed CLP animals relative to water-fed controls failed to achieve significance (Fig 2D).

**Fig 3:**
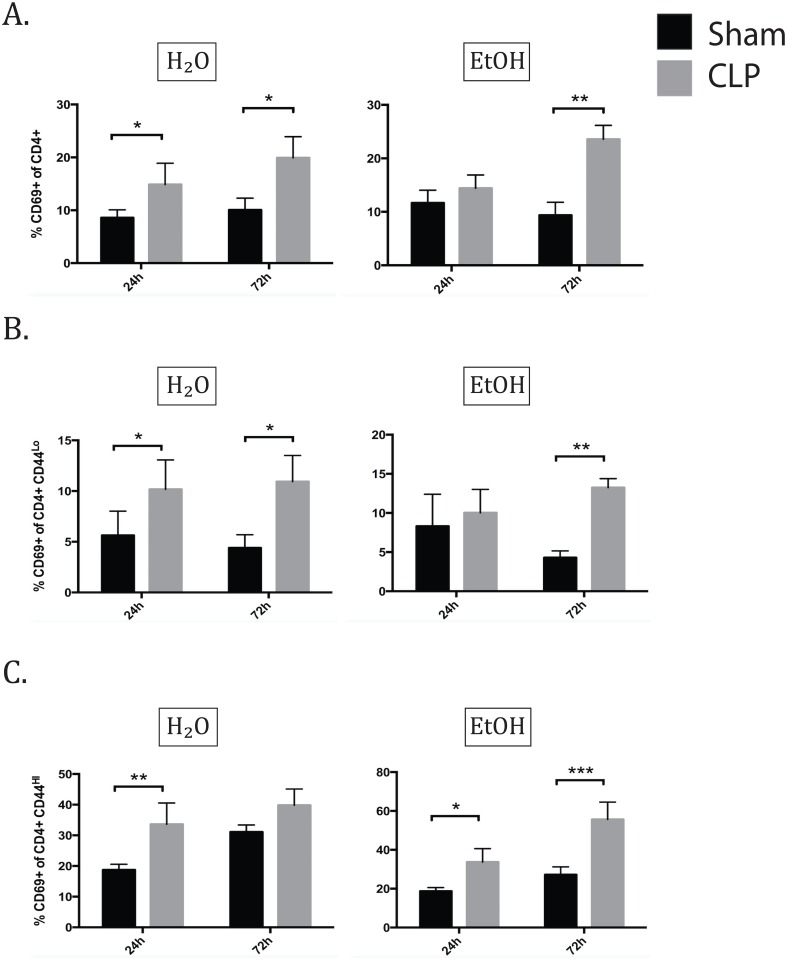
Alcohol delays the kinetics of CD69 expression of naïve CD4+ T cells and prolongs CD69 expression on memory CD4+ T cells. A) At 24h, CD69 expression increased in the water septic group over water sham (11.6±1% vs 8.6±0.6%, p = 0.01). At 72h, CD69 increased due to sepsis in both water and alcohol-fed groups (H2O sham 10.1±0.9% vs H2O CLP 19.9±1.4%, p = 0.04; EtOH sham 9.4±1% vs EtOH CLP 23.6±1.7%, p = 0.004). B) In naïve CD4s at 24h, the water septic group exhibited increase in CD69 expression (10.2±1.1% vs 5.6±1.0%, p = 0.04), while the alcohol fed group did not. By 72h, both alcohol and water fed groups exhibit CD69 upregulation in sepsis (H2O sham 4.4±0.5% vs H2O CLP 10.9±0.9%, p = 0.04; EtOH sham 4.3±0.4% vs EtOH CLP 13.2±0.5%, p = 0.005). C) In memory CD4s at 24h, sepsis increased CD69 in both water and alcohol-fed groups (H2O sham 18.7±0.8% vs H2O CLP 33.6±2.6%, p = 0.006; EtOH sham 23.5±0.9% vs EtOH CLP 36.4±0.8%, p = 0.03). At 72h, CD69 remained increased in alcohol septic mice only (EtOH sham 27.2±1.7% vs and EtOH sepsis 55.6±4%, p = 0.0003). n = 6-9/group.

In the total CD4+ population, CD69 expression increased significantly 24h after sepsis induction in water fed animals. A concomitant significant increase was not seen in alcohol-fed animals at this time point. By 72h, both water and alcohol fed animals demonstrate increased CD69 expression in sepsis compared with sham laparotomy (Fig 3A). In neither group was there a difference between water sepsis and alcohol sepsis in frequency of CD69 positivity, however, this dynamic change in the kinetics of expression suggests that alcohol delays the process of CD4+ T cell activation in sepsis.

We next investigated the expression of CD69 within memory (CD44^hi^) or naïve (CD44^lo^) CD4+ T cell subsets. The pattern of CD69 expression in the CD44^lo^ CD4+ population matched that of the total CD4+ population (Fig 3B). A different pattern was seen in the memory (CD44^hi^) CD4+ T cells, however. Firstly, the memory CD4+ T cells had noticeably higher CD69 expression in the sham groups than the naïve cells. At 24h after sepsis both water and alcohol fed animals significantly increased CD69 expression without a difference between water and alcohol sepsis groups. By 72h, CD69 expression remained increased only the alcohol septic group compared with sham, while the water fed groups were not significantly different (Fig 3C). These data also support delayed activation kinetics of CD4+ CD44^hi^ cells in sepsis following alcohol exposure. In addition to the increased CD69 expression in the sham groups, the alcohol fed subgroup failed to return to baseline CD69 expression by 72h following sepsis.

### Alcohol delayed the increase in frequency of O-glycosylated CD43+ memory CD4+ T cells during sepsis

Given the observation that sepsis in the setting of chronic ethanol exposure resulted in reduced early T cell activation as measured by CD69 expression, we next sought to confirm this finding of delayed T cell activation using another marker associated with differentiation and acquisition of effector function. Specifically, previous studies have shown that expression of the O-glycosylated form of CD43 is indicative of effector status and is associated with potent cytolytic function [25]. O-glycosylated CD43 can be readily detected on the cell surface using an antibody that specifically recognizes this glycoform (clone 1B11). 1B11 binding is low on naïve CD8+ T cells, high on activated CD8+ effector T cells, and is reduced again on quiescent CD8+ memory T cells [25]. Viral immunity studies in CD43-/- mice have shown that CD43 plays a costimulatory role in T cell activation/trafficking as well as a negative regulatory role in the resolution of effector T cell function, indicating a multifaceted role in T cell homeostasis [25]. While T cell memory and activation phenotypes have been described in both alcohol ingestion and sepsis models, the role of CD43 has not to our knowledge been explored in either.

**Fig 4:**
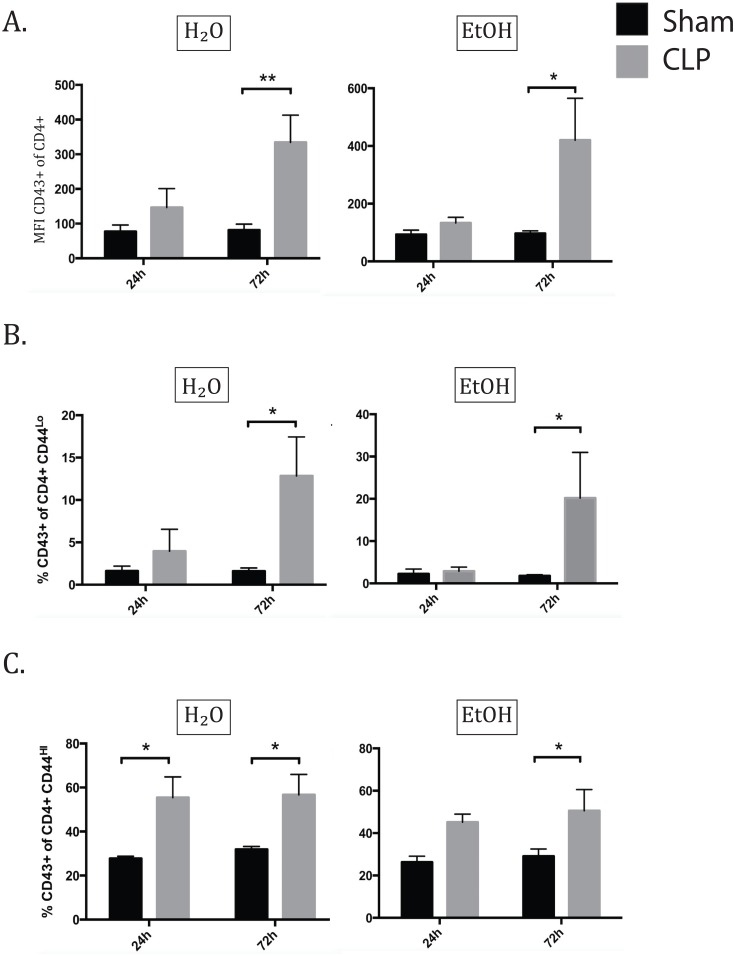
Alcohol delays O-glycosylation of CD43 on memory CD4+ T cells in sepsis. A) At 24h, there was no significant increase in CD43 expression in total CD4 cells in either water or alcohol-fed mice due to sepsis. By 72h, both water sepsis and alcohol sepsis groups increased CD43 expression over shams (H2O sham 81.4±7.0 vs H2O CLP 334.5±27.6, p = 0.004; EtOH sham 96.6±3.7 vs EtOH CLP 420.0±65.0, p = 0.04). B) At 24h, there was no significant increase in CD43 expression in naïve CD4 cells. By 72h, both water sepsis and alcohol sepsis groups increased CD43 expression over shams (H2O sham 1.6±0.4% vs H2O CLP 12.9±1.6%, p = 0.01; EtOH sham 1.7±0.1% vs EtOH CLP 20.2±4.8%, p = 0.01). C) At 24h, water septic mice showed significant upregulation of CD43 on memory CD4s (H2O sham 27.8±0.5% vs H2O CLP 55.4±4.2%, p = 0.02), while alcohol septic mice did not. By 72h, CD43 expression is increased in both water and alcohol sepsis groups compared to sham (H2O sham 31.9±0.5% vs H2O CLP 56.7±3.3%, p = 0.03; EtOH sham 29.1±1.4% vs EtOH CLP 50.6±4.5%, p = 0.02).

In terms of CD43 expression in the overall CD4+ population, ethanol fed sham animals did not demonstrate any baseline change in CD43 expression. 24h following sepsis induction there was no increase in CD43 binding in either water or alcohol fed septic groups. By 72h, both water and alcohol fed groups demonstrated significantly increased MFI of CD43, without a difference between the two septic groups (Fig 4A); this result was the same for frequency of CD43 positivity, but MFI was used to avoid ambiguity in gating. The pattern of CD43 expression in the CD44^lo^ subset matched that of the total CD4+ population. 24h following sepsis, there was no significant difference in CD43 expression due to sepsis in either water or alcohol fed groups. By 72h, both water and alcohol fed septic groups exhibited increased frequencies of CD43+ CD44^lo^ cells as compared to their sham counterparts without a significant difference between the two septic groups (Fig 4B). In the memory CD4+ subset, there was no baseline difference in CD43 expression between sham animals, and 24h following sepsis induction the water-fed septic group demonstrated significantly increased CD43 expression. This effect was abrogated in the presence of alcohol, as the increase in the alcohol fed septic group was not significant at the 24h time point. By 72h, both water and alcohol fed septic groups exhibited significantly increased CD43 expression over sham groups (Fig 4C). These data indicate that there is a delay in the acquisition of O-glycosylated CD43 specifically on CD44^hi^ memory T cells in alcohol sepsis relative to water fed septic controls.

### Alcohol delayed increase in the CD69+ CD43+ population in naïve and memory CD4+ T cells in sepsis

**Fig 5:**
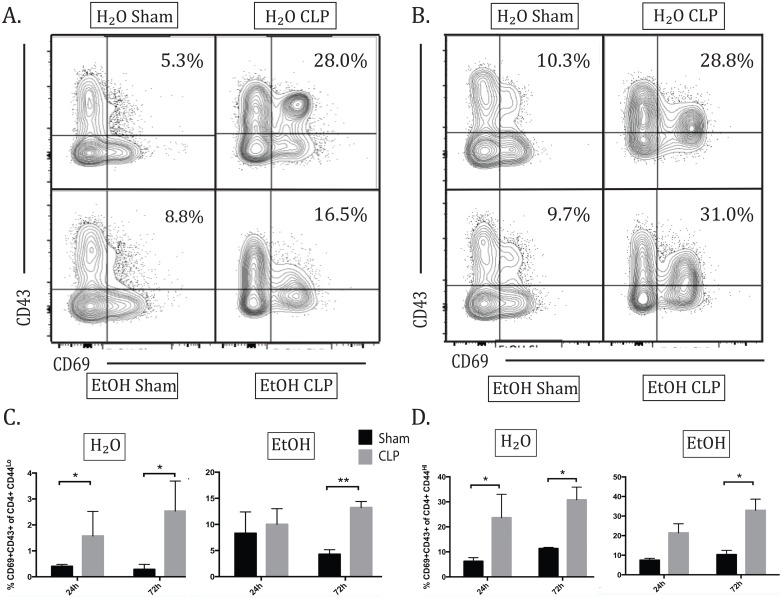
Alcohol delays increase in the CD69+CD43+ population in naïve and memory CD4+ T cells. A) Representative flow plot for frequency of CD69+CD43+ of CD4+ CD44^hi^ at 24h. B) Representative flow plot for frequency of CD69+CD43+ of CD4+ CD44^hi^ at 72h. C) By 24h, there was significant increase in CD69+ CD43+ population in naïve CD4s in water sepsis (H2O sham 0.4±0.04% vs H2O CLP 1.6±0.4%, p = 0.03). This population was not increased in alcohol sepsis. By 72h, both water septic and alcohol septic groups showed significant increase in the CD69+ CD43+ population (H2O sham 0.3±0.08% vs H2O CLP2.5±0.4%, p = 0.02; EtOH sham 0.3±0.05% vs EtOH CLP 3.5±0.5%, p = 0.01). D) In memory CD4s, at 24h there was a significant increase in CD69+ CD43+ population in water sepsis (H2O sham 6.2±0.7% vs H2O CLP 23.7±4.2%, p = 0.02) but not alcohol sepsis. By 72h, both water and alcohol septic groups show significant increase in this population above sham controls (H2O sham 11.4±-.2% vs H2O CLP 30.8±1.8%, p = 0.04; EtOH sham 10.3±0.9% vs EtOH CLP 32.9±2.6%, p = 0.005). n = 4-8/group.

Interestingly, this pattern of delayed CD43 upregulation in alcohol sepsis compared with water sepsis was consistent over subgroup analysis of activated naïve and memory CD4s. Within CD44^hi^ memory CD4+ T cells, a distinct CD69+CD43+ population was identified in the setting of sepsis (Fig 5A). Twenty-four hours after sepsis, there was a significant increase in the frequency of CD69+ CD43+ cells within the CD4+CD44^hi^ subset in water sepsis but not alcohol sepsis. By 72h, both water and alcohol sepsis groups showed significant increase in the frequency of CD44^hi^ CD69+ CD43+ cells above sham controls (Fig 5C). CD69+ CD43+ cells present within the CD44^lo^ compartment also demonstrated the same kinetics as the CD44^hi^ group. Within 24h, there was a significant increase in the frequency of CD69+CD43+ cells within the CD44^lo^ CD4+ T cell population in water sepsis, while the increase in alcohol sepsis failed to reach significance. By 72h, both water septic and alcohol septic groups showed significant increase in the frequency of CD69+CD43+ cells among CD4+ CD44^lo^ cells) (Fig 5D).

### IL-2 production by CD4+ T cells was decreased 72h following sepsis in alcohol-fed animals

**Fig 6:**
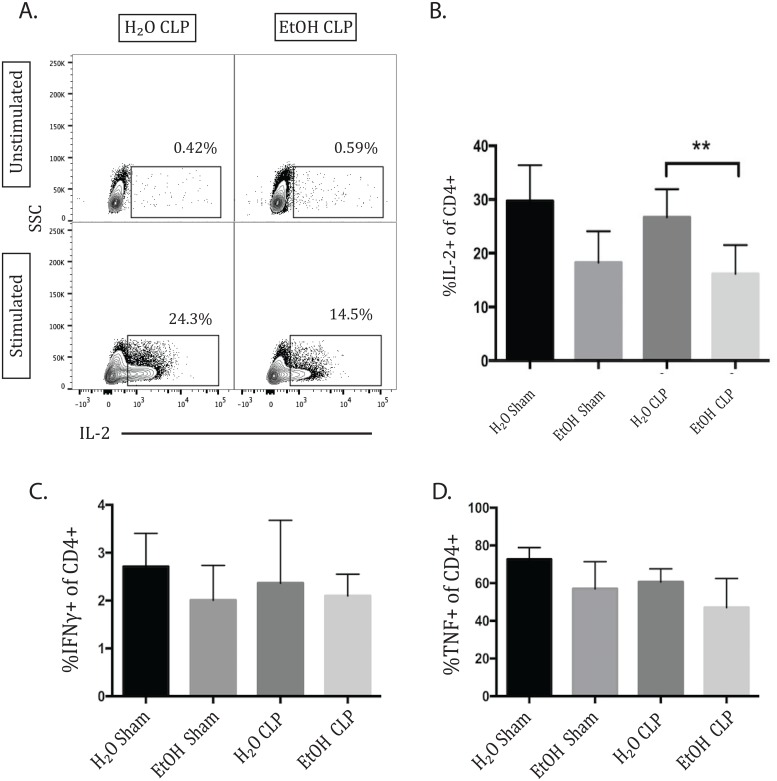
IL-2 (but not IFN-γ or TNF) production by CD4+ T cells is decreased 72h following CLP in alcohol-fed animals. Representative flow plot and summary data 72h following sepsis, demonstrating a trend toward decreased IL-2 production due to alcohol alone between sham groups which did not reach significance, but there was a significant decrease in IL-2 in alcohol septic animals compared with water septic (16.15±1.7% vs 26.7±1.74%, p = 0.004). There were no differences in the frequencies of IFN-γ or TNF- producing CD4+ T cells between any of the groups. n = 7-12/group.

Production of the cytokines IL-2, TNF, and IFN-γ was measured at 24 and 72 hours following CLP or sham laparotomy. Analysis was performed via one-way ANOVA with multiple comparisons test in order to identify changes observed to a greater degree in the combination of sepsis and ethanol than in the presence of each individual variable alone. There was no significant change in CD4+ IL-2 production at 24h (data not shown). At 72h, there was at trend toward decreased IL-2 in the alcohol-fed sham group compared to water-fed sham group, which did not reach significance (p = 0.07). However, there was a significant decrease in IL-2 in alcohol septic animals compared with water septic (Fig 6A and 6B). This demonstrates a more significant reduction in IL-2 in the setting of alcohol sepsis than is seen due to either alcohol or sepsis alone. No differences in TNF or IFN-γ production reached statistical significance (Fig 6C and 6D).

### Alcohol differently affected early activation of naïve and memory CD8+ T cells

**Fig 7:**
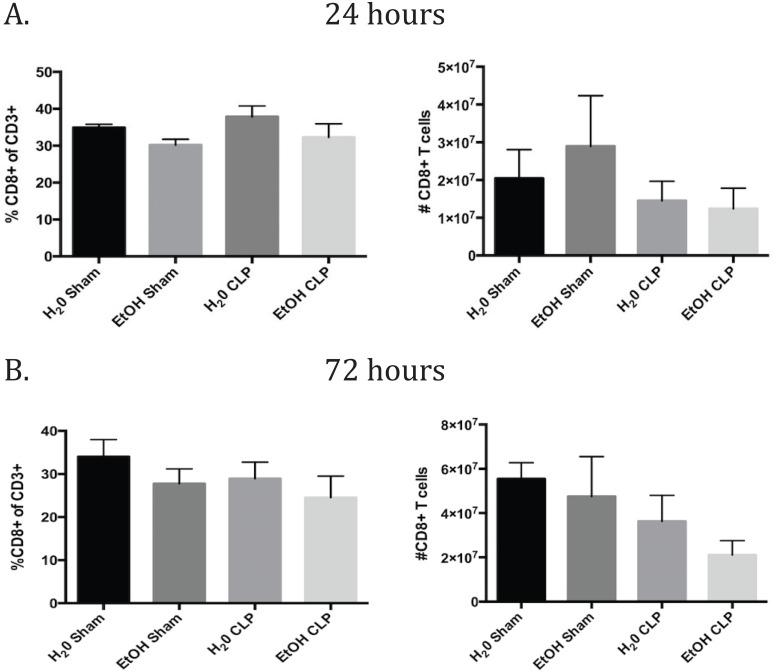
CD8+ T cell frequencies and counts. A) There were no relevant differences in CD8 frequencies or counts specifically due to sepsis or ethanol alone at 24h. B) At 72h, there was a strong trend toward a decrease in absolute count due to sepsis in both the water and alcohol fed groups. n = 6-9/group.

**Fig 8:**
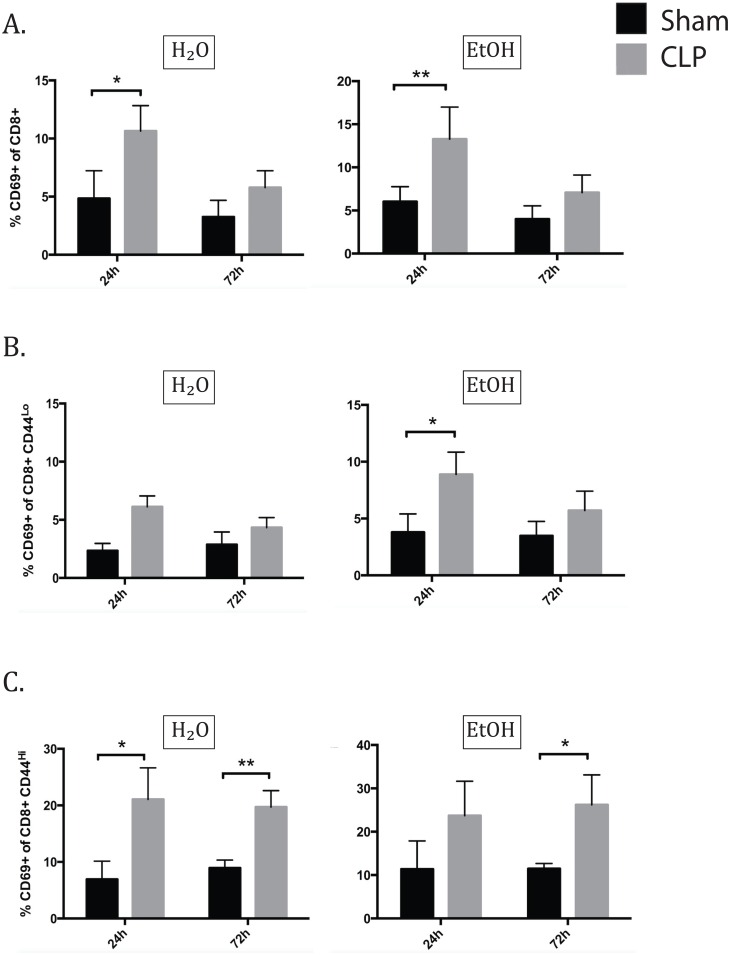
Alcohol differently affects early activation of naïve and memory CD8+ T cells. A) At 24h after CLP, total CD69 expression increased in both the water and alcohol septic groups over sham controls (H2O sham 4.85±1.0% vs H2O CLP 10.65±0.83%, p = 0.03; EtOH sham 6.02±0.71% vs EtOH CLP 13.27±1.24%, p = 0.009). By 72h, the difference in CD69 expression had resolved in both groups. B) In naïve CD8s at 24h, the water septic group exhibited a trend toward increase in CD69 expression but did not reach significance (p = 0.09), while the alcohol fed group did significantly increase CD69 expression (EtOH sham 3.79±0.66% vs EtOH CLP 8.88±0.66%, p = 0.007). By 72h, neither alcohol nor water fed groups exhibit CD69 upregulation. C) In memory CD8s at 24h, sepsis increased CD69 in the water-fed group (H2O sham 6.95±0.57% vs H2O CLP 21.05±%, p = 0.01). The alcohol fed group increased similarly but did not reach significance. At 72h, CD69 remained increased in water septic group (H2O sham 8.93±1.3% vs H2O CLP 19.71±1.03%, p = 0.005) and the increase in the alcohol septic group also was significant (EtOH sham 11.48±0.5% vs and EtOH sepsis 26.2±3.09%, p = 0.02). n = 6-9/group.

In the CD8+ T cell compartment, there were no relevant differences in CD8 frequencies or counts specifically due to sepsis or ethanol alone (Fig 7). In the CD8+ T cell population as a whole 24h after CLP, CD69 expression increased in both the water and alcohol septic groups over sham controls. By 72h, the difference in CD69 expression had resolved in both groups (Fig 8A). Interestingly, the CD44^lo^ and CD44^hi^ subsets demonstrate a very different pattern of CD69 expression. For the CD44^lo^ subgroup at 24h, the water septic group exhibited a trend toward increase in CD69 expression but did not reach significance (p = 0.09), while the alcohol fed group did significantly increase CD69 expression. By 72h, neither alcohol nor water fed groups demonstrated increased CD69 expression (Fig 8B). In the CD44^hi^ subset at 24h, sepsis increased CD69 in the water-fed group. The alcohol fed group increased similarly but did not reach significance. At 72h, CD69 remained increased in water septic group and the increase in the alcohol septic group also was significant (Fig 8C).

### Alcohol decreased the frequency of CD8^dim^ cells in sepsis

**Fig 9:**
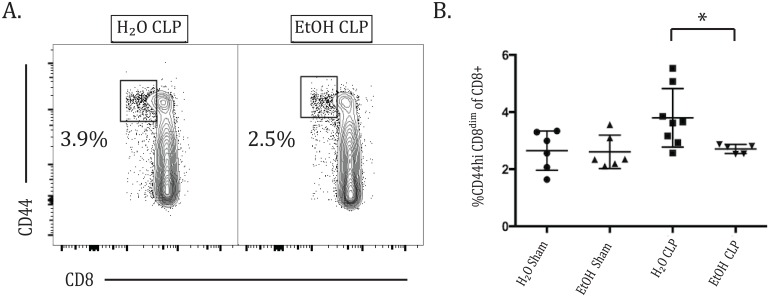
Alcohol decreases the frequency of CD8^dim^ cells in sepsis. A) Contour flow plot of the CD8^dim^ CD44^hi^ population of CD8+ T cells. B) At 72h after CLP, there is an increased frequency of CD8+ CD44^hi^ T cells with low expression of CD8 in water sepsis compared with alcohol sepsis (3.78±0.36% vs 2.71±0.07%, p = 0.01). n = 5-8/group.

It has been previously reported that as CD8+ T cells become activated, they downregulate their CD8 surface expression, resulting in a population of so-called CD8^dim^ cells [26]. In our analyses, we noted that during sepsis a population of CD8^dim^ CD44^hi^ cells could be identified. Interestingly, at 72h after CLP, while this population increased in the water-fed animals, it failed to do so in the alcohol fed group, such that a decreased frequency of CD8^dim^ CD44^hi^ T cells was seen in alcohol sepsis compared with water sepsis, suggesting that alcohol mitigated the emergence of a CD8^dim^ population in sepsis (Fig 9).

### IL-2 production by CD8+ T cells decreased 72h following CLP in alcohol-fed animals

**Fig 10:**
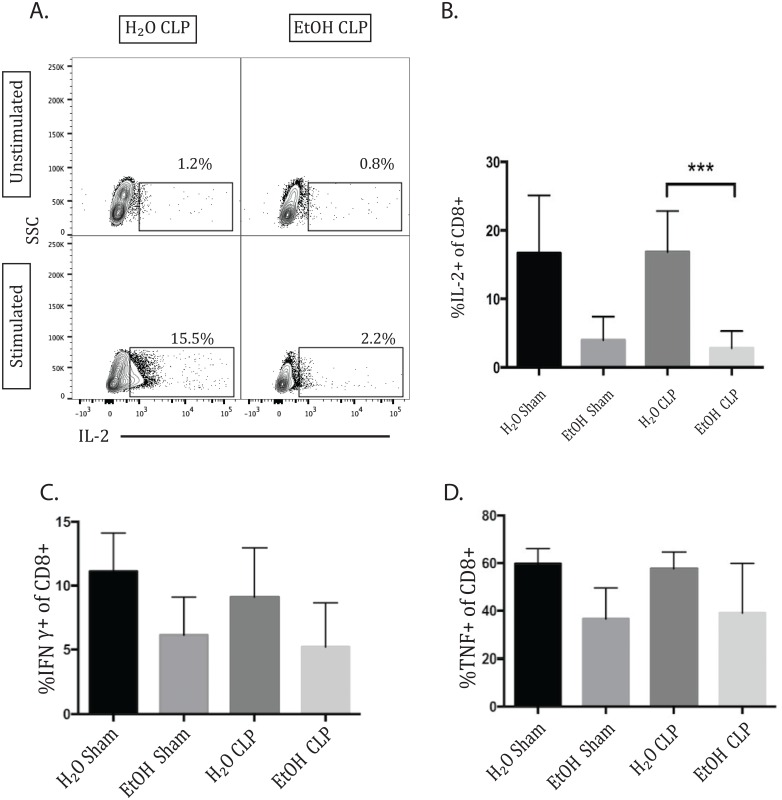
IL-2 (but not IFN-γ or TNF) production by CD8+ T cells is decreased 72h following CLP in alcohol-fed animals. Representative flow plot (A) and summary data (B) 72h after sepsis demonstrating a trend toward decreased IL-2 production due to alcohol alone between sham groups which did not reach significance, and a significant decrease in IL-2 in alcohol septic animals compared with water septic (2.81±0.8% vs 16.87±2.0%, p = 0.0004). There were no differences in the frequencies of IFN-γ or TNF- producing CD8+ T cells between any of the groups. n = 7-12/group.

As with the CD4+ T cell population, CD8+ T cell production of the cytokines IL-2, TNF, and IFN-γ was measured at 24 and 72 hours following CLP or sham laparotomy. No differences in TNF or IFN-γ production reached statistical significance using the multiple comparisons test. There was no significant change in CD8+ IL-2 production at 24h (data not shown). At 72h, there was at trend toward decreased IL-2 production due to alcohol alone between sham groups which did not reach significance (p = 0.1), but there was a significant decrease in IL-2 in alcohol septic animals compared with water septic (Fig 10A and 10B). This demonstrates a more significant reduction in IL-2 in the setting of alcohol sepsis than occurs due to either alcohol or sepsis alone. No differences in TNF or IFN-γ production reached statistical significance (Fig 10C and 10D).

### Analysis of serum cytokines supports Th2 skewing in EtOH septic animals relative to water-fed septic animals

**Fig 11:**
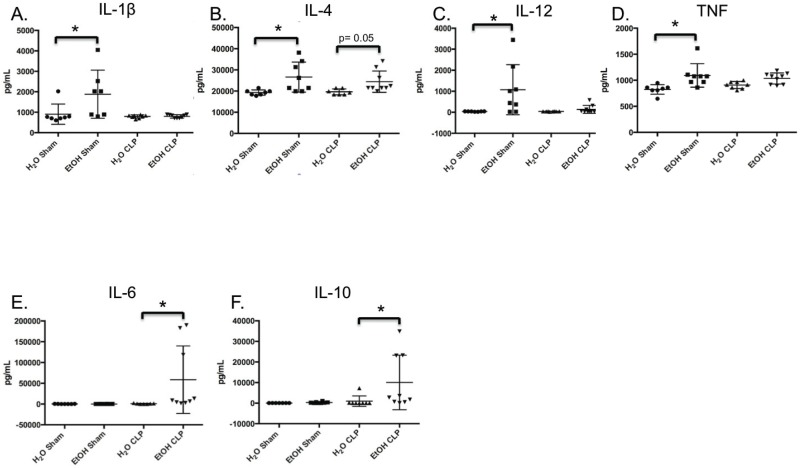
Serum cytokine suggests Th2 skewing in EtOH-fed septic relative to water-fed septic animals. A) Chronic alcohol ingestion induced elevated baseline levels of serum IL1β over water-feeding in sham animals (906.5±187.7 vs 1883±444.6, p = 0.01). B) Alcohol ingestion increased baseline serum IL-4 concentration in sham animals (19322±473.2 vs 26584±2516, p = 0.02), with a concurrent strong trend toward increase in septic animals (19629±523 vs 24433±1686, p = 0.05). C) Chronic alcohol ingestion increased baseline serum IL12 concentration in sham animals (1073±421.5 vs 35.9±3.1, p = 0.04). D) Alcohol ingestion increased baseline serum TNF concentration in sham animals (1093±79.9 vs 823.8±34.7, p = 0.005). E) Serum IL-6 was increased in alcohol sepsis over water sepsis (58697±27081 vs 896.7±356, p = 0.02). F) Serum IL-10 concentration was increased is alcohol sepsis over water sepsis (10057±4412 vs 979.7±896.2, p = 0.01). n = 8/group.

Our data identified reduced frequency of IL-2 secreting cells in both the CD4+ and CD8+ T cell compartments as a functional difference in EtOH-fed septic animals relative to water-fed septic animals. We next sought to determine whether other serum cytokines were different in EtOH-fed vs. water-fed animals following the induction of sepsis. Water-fed or EtOH-fed animals were subjected to CLP, and serum ctyokines were analyzed 24h later. Results indicated a strong trend toward increased serum IL-4 in the EtOH-fed septic relative to water-fed septic animals (p = 0.05, Fig 11B), and a statistically significant increase in both IL-6 and IL-10 in the sera of EtOH-fed septic relative to water-fed septic animals (Fig 11E and 11F). These data suggest that EtOH-fed animals may possess a more Th2 skewed immune response relative to water-fed animals following sepsis.

## Discussion

In this study, we interrogated the impact of chronic alcohol exposure on activation and effector function of CD4+ and CD8+ T cells following sepsis. We identified a pattern of delayed T cell activation and altered effector function in animals chronically exposed to ethanol, relative to water fed controls. These changes were observed across the CD4+ and CD8+ T cell compartments as well as in both naïve and memory T cells. One would expect that in the setting of a polymicrobial septic insult, the CD44^hi^ population of T cells would be a source of rapid recall response to initiate the adaptive immune response, and the fact that we observed a relative delay in the activation kinetics of memory T cells in the setting of chronic alcohol ingestion could be a potential contributor to the observed increase in mortality observed in alcohol fed septic animals. Importantly, we also observed a diminution in the frequency of IL-2 secreting cells in both the CD4+ and CD8+ compartments, and an increase in serum IL-4, IL-6, and IL-10, in the setting of EtOH-CLP relative to H20-CLP (Figs 6 and 10), suggesting that impaired or dysregulated cytokine secretion in the setting of chronic alcohol exposure may underlie observed increased mortality in these animals following CLP [27].

When placed within the context of what is known about the impact of CD43- and CD69-mediated signals on cytokine production, our results further suggest that the observed delayed kinetics of expression of both CD43 and CD69 on CD4+ and CD8+ T cell in alcohol fed animals relative to water-fed animals following CLP may mechanistically underlie the significant alterations in cytokines observed in these animals. First, previously published reports have demonstrated that T cell costimulation through CD43 results in enhanced IL-2 production [28]; conversely, we and others have shown that CD43 deficient T cells exhibit reduced Th1 cytokines (i.e. IL-2, IFN-γ) and increased Th2 cytokines (i.e. IL-4, IL-5) [25, 29–31]. Second, it is known that CD69 signaling results in the activation of STAT5 [32], a critical component of the IL-2 signaling pathway [33]. Because signaling through STAT5 functions in a positive feedback loop to increase both IL-2 production and expression of the IL-2 receptor [33], it is plausible that the reduced CD69 expression on T cells in alcohol-fed septic animals results in reduced STAT5 activation, resulting in decreased engagement of the IL-2 pathway and eventually diminished IL-2 production. This notion is further supported by studies showing that CD69 deficient animals exhibit increased Th17 differentiation [34–36], a process which is antagonized by sufficient availability of ambient IL-2 [37, 38]. Taken together, these data suggest that the reduced CD43 and CD69 expression observed on CD4+ and CD8+ T cells in ethanol-fed animals in the setting of sepsis may be responsible for the dysregulated cytokine response. Given the importance of IL-2 for the survival and proliferation of T cells, we conclude that although T cells from alcohol-fed septic animals do eventually express CD43 and CD69 (by 72h post-CLP), there are important functional defects that arise as a result of this delay.

In addition to their potential role in controlling the acquisition of T cell cytokine function, both CD43 and CD69 have functions outside of their costimulatory role, and thus the altered kinetics in expression of these molecules observed in ethanol-treated animals may also exert effects on immunity during sepsis independent of the observed decrease in IL-2 production. First, CD43 is known to play a critical role in T cell trafficking to draining lymph nodes and areas of inflammation [39] or infection [25]. Thus, the observed delay in upregulation of CD43 during sepsis in alcohol-fed animals could have implications on eradication of septic foci, particularly in the setting of the CLP model, which lacks septic source control. In addition, there are several lines of evidence that suggest that CD43 can also provide a negative regulatory function on T cell activation, proliferation, and adhesion [25, 40]. Thus, further mechanistic investigation into the role of CD43 in sepsis following chronic ethanol ingestion is warranted.

Likewise, while CD69 is also generally regarded as an early marker of T cell activation, there are data implicating it as a counter-regulatory molecule important for the resolution of inflammation [39, 41–44]. High levels of CD69 are found in inflammatory infiltrates in vitro [43], as well as on T cells of critically ill patients with sepsis relative to non-septic controls [45]. However, in animal models, CD69 deficiency resulted in counterintuitive increases in proinflammatory responses in animal models such as collagen-induced arthritis [43] and Th2-mediated asthma [41], suggesting that this pathway may function as a negative regulator of inflammation. Thus, the prolongation of CD69 expression in the memory CD4+ population we observed on alcohol-fed septic animals may indicate dysregulation of this T cell subset by lack of appropriate counter-regulation.

It has been previously reported in the literature that chronic ethanol ingestion is associated with increased CD44 expression in CD4+ and CD8+ T following in vitro T cell receptor stimulation by immobilized anti-CD3 antibody [10]. Interestingly, we did not observe any increase in the frequency of CD44 on CD8+ T cells due to ethanol alone or following sepsis. We postulate that this difference between in vitro simulation and CLP could be indicative of the widespread dysregulation of the immune response increasingly recognized in sepsis [13–15, 19]. Furthermore, we identified a population of CD8^dim^CD44^hi^ cells that emerged during sepsis which was significantly decreased in alcohol fed animals compared with water-fed animals 72 hours after CLP. Several published reports [26, 46] suggest that these may be antigen-specific CD8+ T cells that have downregulated the CD8 co-receptor following TCR ligation, and if so these findings would indicate a delay in expansion of antigen-specific CD8+ T cells following sepsis in the setting of chronic alcohol exposure. Future experiments using tools to track antigen-specific cell responses are likely warranted to further elucidate this relationship. It is also important to note that in this study there was a strong trend toward decreased absolute CD4^+^ T cell count in the water sham compared to water-CLP group hours (p = 0.2), however it did not achieve statistical significance as has been found in other reports on the immunologic effects of sepsis [47]. We attribute this more to the variability in the model than to an actual qualitative difference in our findings relative to previously published reports; the factors that impact the degree of CD4+ T cell apoptosis vs. survival following CLP warrant further investigation.

In sum, it is likely that the qualitative changes in T cell kinetics we observed in the setting of alcohol sepsis are part of a much larger, multifaceted network of immunological changes, encompassing both the innate and adaptive arms of the immune system. As investigation into immunomodulatory therapies for sepsis progresses, the data presented here may encourage consideration of subgroup analysis of chronic ethanol users, as benefit may be differently demonstrated in this unique immunological environment.
